# Foot-and-Mouth Disease in the Middle East Caused by an A/ASIA/G-VII Virus Lineage, 2015–2016

**DOI:** 10.3201/eid2406.170715

**Published:** 2018-06

**Authors:** Katarzyna Bachanek-Bankowska, Antonello Di Nardo, Jemma Wadsworth, Elisabeth K.M. Henry, Ünal Parlak, Anna Timina, Alexey Mischenko, Ibrahim Ahmad Qasim, Darab Abdollahi, Munawar Sultana, M. Anwar Hossain, Donald P. King, Nick J. Knowles

**Affiliations:** The Pirbright Institute, Woking, UK (K. Bachanek-Bankowska, A. Di Nardo, J. Wadsworth, E.K.M. Henry, D.P. King, N.J. Knowles);; Foot-and-Mouth Disease Institute, Ankara, Turkey (Ü. Parlak);; Federal Centre for Animal Health, Vladimir, Russia (A. Timina, A. Mischenko);; Ministry of Environment, Water and Agriculture, Riyadh, Saudi Arabia (I.A. Qasim);; Iran Veterinary Organization, Tehran, Iran (D. Abdollahi);; University of Dhaka, Dhaka, Bangladesh (M. Sultana, M.A. Hossain)

**Keywords:** foot-and-mouth disease, FMD, outbreaks, foot-and-mouth disease virus, FMDV, aphthovirus, viruses, A/ASIA/G-VII virus lineage, epidemiology, vaccination, cattle, zoonoses, vaccines, Indian subcontinent, Middle East

## Abstract

Phylogenetic analyses of foot-and-mouth disease type A viruses in the Middle East during 2015–2016 identified viruses belonging to the A/ASIA/G-VII lineage, which originated in the Indian subcontinent. Changes in a critical antigenic site within capsid viral protein 1 suggest possible evolutionary pressure caused by an intensive vaccination program.

Foot-and-mouth disease (FMD) can decrease productivity in the cloven-hooved livestock industry. As this disease spreads rapidly over large distances, it is regarded as one of the most economically devastating diseases of livestock. FMD is caused by FMD virus (FMDV; family *Picornaviridae*, genus *Aphthovirus*), which has 7 immunologically distinct serotypes, O, A, C, Asia 1, SAT 1, SAT 2, and SAT 3. Worldwide, ecologic niches of FMDV circulation have been defined as 7 virus pools. Pools 1–3 are present in Asia, where only serotypes O, A, and Asia 1 are present.

Serotype A viruses are considered to be the most variable (genetically and antigenically) of Eurasian serotypes. Three topotypes (ASIA, AFRICA, and EURO-SA [Europe–South America]) and multiple diverse lineages and sublineages have been identified (*1*). The ASIA topotype is widespread and is found in most countries in Asia; there have been sporadic incursions into North Africa. Although the G-VII lineage (also known as genotype 18) (*2**,**3*) usually circulates in countries containing virus pool 2 (commonly in Bangladesh and India, rarely in Bhutan and Nepal, but until now not in Sri Lanka), this lineage has also been reported in Saudi Arabia in 1995, Albania and the former Yugoslav Republic of Macedonia in 1996, and Myanmar in 2010.

In India, viruses of the A/ASIA/G-VII lineage were isolated in 1983 (*4*) and until 2001 were co-circulating with the A/ASIA/G-VI lineage (*2*). After 2001, only the G-VII lineage has been reported (*5*). Overall incidence of FMD outbreaks caused by serotype A in India during 2011–2016 was low (3.1% of the total reported outbreaks). During the same time, 86.8% of outbreaks were caused by serotype O, and 10.1% by serotype Asia 1 (*6*).

Despite the low number of outbreaks investigated, emergence of a capsid viral protein (VP) 3 deletion variant, VP^59^, was reported in the A/ASIA/G-VII lineage during 2002 (*4*). Currently, a group of viruses described as clade C, a subgroup within the VP3^59^-deletion variant, is speculated to be the dominant group of the A/ASIA/G-VII lineage prevalent in India (*2*). We report foot-and-mouth disease in the Middle East during 2015–2016 caused by an A/ASIA/G-VII virus lineage.

## The Study

During outbreak investigations of FMD in Saudi Arabia in 2015, a virus of the A/ASIA/G-VII lineage (VP3^59^-deletion variant), was identified in cattle (*7*). The outbreak spread quickly to several strictly monitored dairy farms that had high rates of vaccination, as well as to nomadic herds. Concurrently, related viruses were found in Armenia, Iran, and Turkey in 2015 and continued to circulate in Saudi Arabia, Turkey, and Iran in 2016. Sequences of VP1-coding regions from samples submitted to the Food and Agriculture Organization of the United Nations World Reference Laboratory for FMD (Pirbright, UK) were determined by using described methods (*8*). VP1 sequences from outbreaks in Armenia, Bangladesh, and Turkey were determined at the Federal Centre for Animal Health (Vladimir, Russia); the University of Dhaka (Dhaka, Bangladesh); and the Foot-and-Mouth Disease Institute (Ankara, Turkey), respectively (Table).

**Table:** Characteristics of 57 strains **of** foot-and-mouth disease viruses used in analysis of foot-and-mouth disease caused by an A/ASIA/G-VII virus lineage, Middle East, 2015–2016

Virus designation	Country	Location	Species	Date collected	GenBank accession no.
A/ARM/1/2015*	Armenia	Armavir, Arazap	Cattle	2015 Dec 25	KY982279
A/ARM/2/2015*	Armenia	Armavir, Arazap	Cattle	2015 Dec 25	KY982280
A/ARM/3/2015*	Armenia	Armavir, Arazap	Cattle	2015 Dec 25	KY982281
BAN/CH/Sa-304/2016	Bangladesh	Chittagong	Cattle	2016 Sep 27	KY077630
A/IRN/8/2015	Iran	Qom	Cattle	2015 Aug 30	KY982282
A/IRN/12/2015	Iran	Qom	Cattle	2015 Sep 9	KY982283
A/IRN/13/2015	Iran	Qom	Cattle	2015 Sep 28	KY982284
A/IRN/14/2015	Iran	Qom	Cattle	2015 Sep 28	KY982285
A/IRN/17/2015	Iran	Qom	Cattle	2015 Oct 9	KY982286
A/IRN/18/2015	Iran	Tehran	Cattle	2015 Oct 10	KY982287
A/IRN/21/2015	Iran	Qom	Cattle	2015 Oct 24	KY982288
A/IRN/22/2015	Iran	Tehran	Cattle	2015 Oct 24	KY982289
A/IRN/25/2015	Iran	East Azerbaijan	Cattle	2015 Oct 28	KY982290
A/IRN/27/2015	Iran	Kermanshah	Cattle	2015 Nov 8	KY982291
A/IRN/1/2016	Iran	Qom	Cattle	2016 Jan 4	KY982292
A/IRN/8/2016	Iran	Tehran	Cattle	2016 Feb 4	KY982293
A/IRN/11/2016	Iran	Qazvin	Cattle	2016 Feb 27	KY982294
A/IRN/12/2016	Iran	Ardebil	Cattle	2016 Feb 29	KY982295
A/IRN/20/2016	Iran	Yazd	Cattle	2016 Feb 4	KY982296
A/IRN/23/2016	Iran	Alborz	Cattle	2016 Apr 7	KY982297
A/SAU/1/2015	Saudi Arabia	Farm A, Durma	Cattle	2015 Sep 2	KU127247
A/SAU/2/2015	Saudi Arabia	Farm A, Durma	Cattle	2015 Sep 2	KY982298
A/SAU/3/2015	Saudi Arabia	Farm B, Al Kharj	Cattle	2015 Oct 9	KY982299
A/SAU/4/2015	Saudi Arabia	Farm B, Al Kharj	Cattle	2015 Oct 19	KY982300
A/SAU/5/2015	Saudi Arabia	Farm C, Al Kharj	Cattle	2015 Oct 16	KY982301
A/SAU/6/2015	Saudi Arabia	Farm A, Durma	Cattle	2015 Oct 21	KY982302
A/SAU/7/2015	Saudi Arabia	Farm D, Al Kharj	Cattle	2015 Oct 23	KY982303
A/SAU/8/2015	Saudi Arabia	Farm D, Al Kharj	Cattle	2015 Dec 30	KY982304
A/SAU/9/2015	Saudi Arabia	Al Kharj	Cattle	2015 Oct 5	KY982305
A/SAU/14/2015	Saudi Arabia	Al Kharj	Sheep	2015 Oct 26	KY982306
A/SAU/15/2015	Saudi Arabia	Al Kharj	Sheep	2015 Oct 26	KY982307
A/SAU/16/2015	Saudi Arabia	Al Kharj	Sheep	2015 Oct 26	KY982308
A/SAU/17/2015	Saudi Arabia	Al Kharj	Sheep	2015 Oct 26	KY982309
A/SAU/21/2015	Saudi Arabia	Al Kharj	Cattle	2015 Dec 22	KY982310
A/SAU/15/2016	Saudi Arabia	Farm D, Al Kharj	Cattle	2016 Mar 27	KY982311
A/SAU/19/2016	Saudi Arabia	Farm C, Al Kharj	Cattle	2016 Oct 14	KY982312
A/SAU/20/2016	Saudi Arabia	Farm C, Al Kharj	Cattle	2016 Oct 14	KY982313
A/SAU/21/2016	Saudi Arabia	Mekkah	Cattle	2016 Oct 19	KY982314
A/SAU/22/2016	Saudi Arabia	Mekkah	Cattle	2016 Oct 19	KY982315
A/SAU/24/2016	Saudi Arabia	Mekkah	Cattle	2016 Oct 19	KY982316
A/SAU/37/2016	Saudi Arabia	Al Kharj	Cattle	2016 Dec 29	KY982317
A/SAU/40/2016	Saudi Arabia	Al Kharj	Cattle	2016 Dec 29	KY982318
A/SAU/41/2016	Saudi Arabia	Al Kharj	Cattle	2016 Dec 29	KY982319
A/SAU/42/2016	Saudi Arabia	Al Kharj	Cattle	2016 Dec 29	KY982320
A/TUR/175/2015.712*	Turkey	Van	Cattle	2015 Sep 29	KY982321
A/TUR/198/2015.808*	Turkey	Van	Cattle	2015 Oct 15	KY982322
A/TUR/203/2015.827*	Turkey	Van	Cattle	2015 Oct 22	KY982323
A/TUR/219/2015.865*	Turkey	Düzce	Cattle	2015 Nov 10	KY982324
A/TUR/305/2015.923*	Turkey	Yozgat	Cattle	2015 Nov 24	KY982325
A/TUR/331/2015.923*	Turkey	Kütahya	Cattle	2015 Nov 27	KY982326
A/TUR/48/2016.019*	Turkey	Iğdir	Sheep	2016 Jan 8	KY982327
A/TUR/1008/2016.500*	Turkey	Muş	Cattle	2016 Jun 29	KY982328
A/TUR/1193/2016.731*	Turkey	Kastamonu	Cattle	2016 Sep 21	KY982329
A/TUR/1210/2016.750*	Turkey	Kars	Cattle	2016 Sep 26	KY982330
A/TUR/1218/2016.769*	Turkey	Tokat	Cattle	2016 Oct 3	KY982331
A/TUR/1225/2016.769*	Turkey	Gümüşhane	Cattle	2016 Oct 6	KY982332
A/TUR/1227/2016.750*	Turkey	Ardahan	Cattle	2016 Sep 30	KY982333

*Non–World Reference Laboratory for Foot-and-Mouth Disease (Pirbright, UK) reference number.

**Figure 1:**
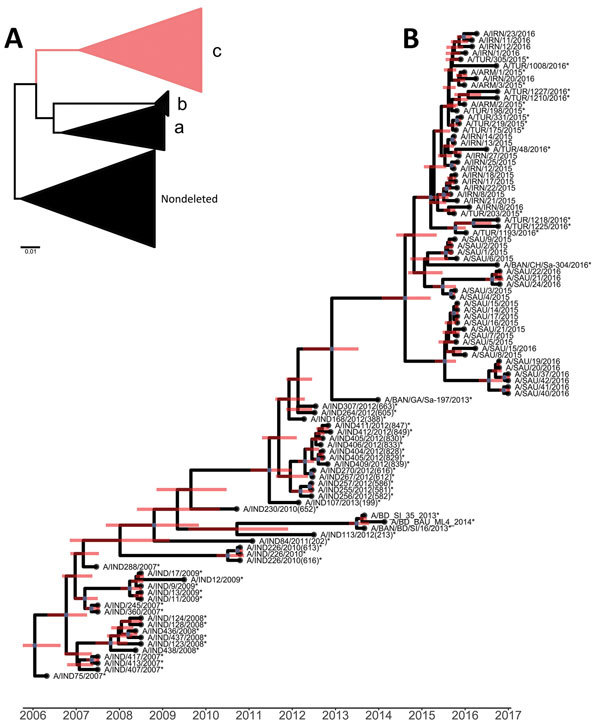
Phylogenetic analyses of viral protein 1–coding sequences of foot-and-mouth disease viruses classified within the A/ASIA/G-VII lineage (isolated during 2006–2017) and reference viruses. A) Maximum-likelihood analysis showing 4 clades (sublineages). Scale bar indicates nucleotide substitutions per site. B) Bayesian phylogenetic analysis of viruses grouping within clade C. Red lines indicate 95% high posterior density of the most recent common ancestor. *Non–World Reference Laboratory for Foot-and-Mouth Disease (Pirbright, UK) reference number. ARM, Armenia; BAN, Bangladesh; IND, India; IRN, Iran; SAU, Saudi Arabia; TUR, Turkey.

We performed maximum-likelihood analyses to compare VP1 coding sequences with other contemporary sequences of the A/ASIA/G-VII lineage and grouped them within the VP3^59^-deleted C clade (*2*) (Figure 1, panel A). We estimated time-resolved phylogenetic trees for 101 serotype A FMDV G-VII VP1 sequences by using BEAST version 1.8.4 (*9*) and incorporated the general time-reversible model with gamma-distributed rate variation among sites and 0.5 prior proportion of invariant sites, the Bayesian Skyline tree before accounting for demographic uncertainty, and a log-normal uncorrelated relaxed clock across branches (*10*). We ran Markov Chain Monte Carlo analysis for 200 million steps and sampled trees every 20,000 steps after a burn-in of 20 million steps. We assessed convergence and good mixing of the Markov Chain Monte Carlo C chain by using Tracer version 1.6 (http://beast.community/tracer).

VP1 coding region–based Bayesian analyses identified >2 independent introductions of G-VII virus into the study region, >1 to Saudi Arabia and 1 to Iran (Figure 1, panel B). However, lack of sequences for recent viruses circulating in the Indian subcontinent makes it difficult to resolve more precisely the number of introductions. Closely related viruses might be circulating in a wider geographic area, thus being a source of the outbreaks. However, this speculation is not supported by available epidemiologic information.

Outbreaks in Armenia, Iran, and Turkey were closely related and most likely originated from the same source. The most recent common ancestor (MRCA) was dated to March 2015 (95% high posterior density [HPD] October 2014–July 2015). The MRCA of the C clade was dated to January 2006 (95% HPD June 2005–April 2006), which is consistent with the first isolate obtained in India during 2007 (*2*). The MRCA of the phylogenetic cluster grouping Middle East (Saudi Arabia, Iran, and Turkey) isolates was dated to July 2015 (95% HPD April–October 2015). Potential movement of G-VII FMDV lineages from the Indian subcontinent might be dated to October 2014 (95% HPD January 2014–February 2015). Evolution of the G-VII C clade lineage was estimated as having a mutation rate of 1.1 × 10^−2^ nt/site/y (95% HPD 8.0 × 10^−3^ to 1.4 × 10^−2^ nt/site/y).

**Figure 2:**
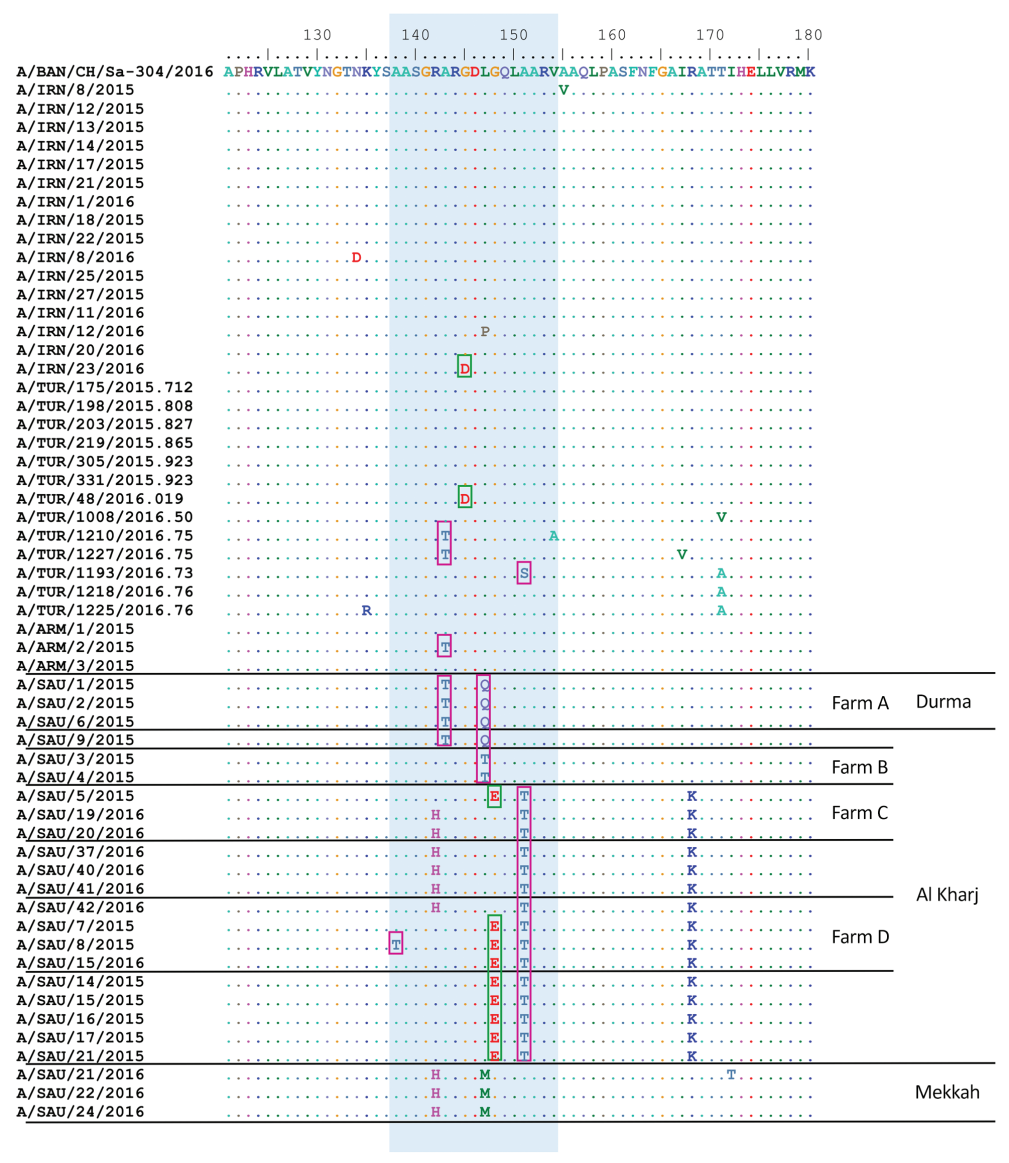
Comparison of predicted amino acid sequences of foot-and-mouth disease viruses showing changes in major antigenic sites. Predicted amino acid sequences for samples collected during outbreaks of foot-and-mouth disease during 2015–2016 were compared with A/BAN/CH/Sa-304/2016 virus sequence. Blue shading indicates conservative changes within antigenic site 1, pink boxes indicate hydrophobic to hydrophilic substitutions, and green boxes indicate hydrophobic to acidic substitutions. Dots indicate sequence identity. Amino acid residues are colored according to their physicochemical properties. Four large dairy farms (containing >1,000 lactating cows), which were multiply sampled, are indicated. ARM, Armenia; BAN, Bangladesh; IRN, Iran; SAU, Saudi Arabia; TUR, Turkey.

Amino acid substitutions on the FMDV surface, particularly in the G-H loop of VP1 (antigenic site 1), have been implicated in antigenic variation of the virus in vitro and in vivo (*11*). Predicted amino acid sequences obtained from samples collected during outbreaks in 2015–2016 were compared with the A/BAN/CH/Sa-304/2016 sequence (most closely related virus from the Indian subcontinent), and 8 nonconservative substitutions were identified at 6 positions (138, 143, 145, 147, 148, and 151) within the VP1 antigenic site 1 (*12**,**13*) (Figure 2; Table).

Changes from hydrophobic (alanine and leucine) to hydrophilic (threonine, glutamine, and serine) amino acid residues were most common, found at 4 positions; changes from hydrophobic (glycine) to acidic (aspartic acid and glutamic acid) amino acids were found at 2 positions. Antigenic variation at site 1 of type C viruses is often based on alternate switching between alanine and threonine residues without accumulation of amino acid substitutions (*14*). In addition, 2 independent changes that did not alter the amino acid characteristics were identified at positions 142 and 147. We also showed that changes at antigenic site 1 were conserved mainly within but differed between farms, supporting independent selection pressures. Although the same vaccine was used, the intensive and frequent vaccination regimen routinely used on the affected farms in Saudi Arabia might have led to an independent antigenic evolution on an individual farm level from chance substitutions. Nevertheless, occasional substitutions within antigenic site 1 were also observed in Armenia, Turkey, and Iran (Figure 2).

## Conclusions

As reported for the O/ME-SA/Ind-2001d virus lineage (*15*), A/ASIA/G-VII is the second FMDV lineage believed to have originated in the Indian subcontinent since 2013 and resulted in extensive outbreaks outside its usual area of distribution. Similar to the Ind-2001d outbreaks, current outbreaks caused by the G-VII lineage appear to be linked to multiple introductions of the virus from the Indian subcontinent; the virus then spread among susceptible ruminant populations in Saudi Arabia, Iran, Turkey, and Armenia.

It is a concern that in vitro vaccine matching data (by virus neutralization) provide poor confidence that commercially available vaccines would offer effective protection against the G-VII lineage (A. Ludi, pers. comm., May 2016). To improve control programs, it is crucial to identify expected routes of FMDV escape (e.g., international trade in animals and animal products) outside historically defined geographic distribution, and to establish transmission pathways within affected areas. To reconstruct likely transmission pathways at greater resolution, genome sequencing of viruses described in this report is currently in progress.
